# Identifying factors affecting willingness to participate in floating population health volunteer services by Chinese volunteers based on the theory of the planned behavior expansion model

**DOI:** 10.3389/fpsyg.2022.953575

**Published:** 2022-10-05

**Authors:** Wei-ling Wu, Hai-Yan Yu, Hai-Xia Zhou

**Affiliations:** ^1^School of Medicine, Guizhou University, Guizhou, China; ^2^School of Public Health Management, Wenzhou Medical University, Wenzhou, China

**Keywords:** floating population, health volunteer service, planned behavior theory, Chinese volunteers, willing

## Abstract

China has the world's largest internal migrant population, called the floating population. Compared to local residents, the floating population utilizes different health services and relies heavily on health volunteer services for supplementary services. In this study, the theory of planned behavior model was used to study the willingness of volunteers to participate in floating population health volunteer services. We examined the effects of several factors on willingness to participate and found that attitude and subjective norm, but not perceived behavioral control, have significant predictive effects on willingness to participate in health volunteer services. Furthermore, altruistic values, social incentives, and personality traits not only have significant predictive effects on volunteer participation but also indirectly affect willingness through attitude and subjective norms. These findings help us understand what factors affect volunteers' willingness to provide health services to the floating population and have important implications for mobilizing volunteers for floating population health services.

## Introduction

In 2019, the global international migrant population was 272 million, an increase of 51 million from 2010 (United Nations Department of Economics Social Affairs/Population Division, 2019). International immigration mainly occurs because of the gap between national income and living standards (Martinez et al., 2015). Recently, China has experienced a wave of internal migrants known as the floating population. The *2019 Chinese Floating Population Development Report* published by the National Health Commission showed that the total floating population in China last year was rooted in economic hardships and comprised 244 million people (National Health Family Planning Commission of China., 2019). Notably, internal migrants who are able to provide documented proof of local identification, known as the household register, receive governmental health service benefits, while those without household registration do not (Liang, 2004).

The health of immigrants has long been a key issue in international public health (Heide et al., 2015), and residential isolation, poor working environments, and social discrimination contribute to the physical and mental health issues experienced by immigrants (Zhang, 2009). Compared to non-immigrants, the physical health status of first-generation Asian immigrants in the United States is poor (Lam and Yip, 2012). Furthermore, a large proportion of Cambodian refugees in the US suffer from mental disorders (Takeuchi et al., 2007); in Europe, the risk of depression in immigrants is 1.6 times higher than in local residents (Ladin, 2013). This difference is directly related to the unequal treatment of immigrants in health service utilization (Wang and Do, 2018). Compared to people of the same age born in the US, undocumented Latin American immigrants not only have a lower health service utilization rate (Ortega et al., 2007) but are clearly excluded from medical subsidies (Sommers, 2013). Although China's floating population and international immigrants are located in different regions, both groups face similar health and health service utilization problems. Indeed, China's floating population faces both poor living environments and lower health literacy compared to other Chinese populations (Zhu, 2014). While local health services were designed to provide solutions for maintaining and improving the health of the floating population, the proportion of health services utilized is far less in the floating population than in populations with local household registration.

In 2017, the floating population service center of the National Health Commission conducted a survey of factors influencing health, health services utilization, and associated illness in 13,998 individuals from the floating population and 14,000 individuals with household registration in Suzhou, Qingdao, Zhengzhou, Guangzhou, Changsha, Xishuangbanna Dai Autonomous Prefecture, and Urumqi (National Health Family Planning Commission of China, 2017). In the current study, we aimed to analyze the results of this survey. While medical records were created for 31.5% of the floating population and 60.5% of local residents, only 39.4% of the floating population utilized community health services compared to local residents (65.6%). Furthermore, due to restrictions in China's unique household registration system, medical insurance for the floating population is not flexible and created additional barriers for individuals attempting to utilize health services (Zhao et al., 2014). Finally, eastern China lacks the medical resources necessary to satisfy the healthcare needs of the floating population (Cai and Yang, 2019) and supplementation by a third department is often required. Hence, health volunteer services are frequently utilized to apply resources to the floating population and compensate for deficiencies in governmental supply capabilities (Salamon, 1999).

Volunteer services have been utilized in various healthcare domains since the announcement of the Alma-Ata Protocol (Akintola, 2011). Notably, volunteers have aided in the healthcare of the elderly (Tang, 2009), provided hotlines for mental health support (Sundram et al., 2018), allocated specialized pediatric palliative treatment (Burbeck et al., 2015), and implemented ambulatory services (Xu, 2008). Although China's volunteer services program is still in its infancy, the service domains are continuously expanding. Indeed, volunteer services have started to provide aid to the floating population, although the mobilization of more volunteers would be beneficial.

In the current study, we aimed to (1) examine how to effectively recruit volunteers to participate in floating population health volunteer services and (2) determine what factors affect volunteer participation in health volunteer services. Behavioral Decision Theory, in which behavioral intention serves as an antecedent for behavioral decision, provides a reference for analyzing these questions. Here, we utilized the Theory of Planned Behavior (TPB) to study the willingness of medically oriented volunteers to participate in floating population health volunteer services. TPB utilizes three factors (attitude, subjective norm, and perceived behavioral control) to determine the behavioral intention of an individual (Duan, 2008). Previously used in studies of physical exercise, garbage sorting, job seeking, consumption purchase intention, travel volunteer services, and other domains (Hobbs, 2008; Akman, 2014; Witzling and Shaw, 2015), TPB has been well-validated and was employed here to study the factors affecting willingness to participate in floating population health volunteer services. Notably, as behavioral intention is determined by the interactions between intrinsic motivation and the external environment (Marsden et al., 2015), we added three factors (altruistic values, personality traits, and social incentives) to the TPB model in this study to complement the three TPB factors previously mentioned. By determining the factors affecting the willingness of individuals to volunteer, we hope to design measures to encourage more professionals to participate in health volunteer services. Indeed, the continued willingness of individuals to volunteer will aid in reducing the health problems of internal immigrants in China and provide a valuable reference for solving similar issues in international immigrant populations.

### Hypotheses

Volunteer service is not only beneficial to society but can also improve the happiness, life satisfaction, self-esteem, physical health, and tendency toward depression in the volunteers themselves (Thoits, 2001). Altruistic self-interest is the main paradigm for analyzing motivation to volunteer. However, this paradigm is a *post hoc* explanation and not a motivational mechanism. Since the intention to volunteer is an outcome of the interaction between individual psychology and social environment, associated influencing factors must be extracted from these two constructs. Widely used in behavioral science studies, TPB is the theoretical foundation for studying the effects of subjective psychological factors on individual behavior. In addition to variables, such as attitude, subjective norm, and perceived behavioral control in TPB, studies have also demonstrated the importance of including other variables that expand upon the existing model including values, personality traits, and incentives (Kim and Cha, 2010; Dowd, 2013). Psychological processes are often divided into three stages: awareness (the information gathering stage), emotion (the innate positive or negative response), and will (the analysis of obstacles; Escalas, 2007). Planned behavioral elements (including personality traits and altruistic values) act on these three stages. For example, dedication consciousness and personality traits affect awareness; altruistic values and attitudes affect emotional responses; and subjective norms and perceived behavioral control affect the will. Furthermore, social incentives often affect individual psychology, while external material and spiritual incentives appear to have regulating effects on health volunteer services, that is, the reduction of cost-associated worries and the social affirmation of the value of volunteering.

### Theory of planned behavior

The theory of planned behavior states that behavioral intention is affected by attitude, subjective norms, and perceived behavioral control (Ajzen and Thomas, 1986). Academic research on TPB and volunteer services generally encompasses two areas: the first attempts to validate the performance of TPB in revealing the behavioral intention for volunteer services, and the second aims to identify new explanatory variables based on the TPB model. Indeed, a prospective study on 81 elderly volunteers in Australia compared the prediction performance of TPB and the volunteer functions inventory. Regression analysis showed that the TPB performed better than the volunteer functions inventory (Greenslade, 2005). Notably, TPB has been effective in explaining a volunteer's intention to participate in health volunteer services: the planned behavior scale accurately demonstrated the intention of nurses to provide volunteer services to SARS patients (Tiraieyari, 2018). Similarly, the TPB model has been used to predict the intention of students to participate in community volunteer services. In these studies, results showed that attitude, normative influences, control perceptions, moral obligation, and past behavior explain 67% of intention differences (Hyde, 2013; Ling, 2016; Lee, 2017). Furthermore, the TPB model has also been used in the study of payment method selection. Radic et al. (2022) explored the problem of tourists adopting CBDC as their payment method by focusing on attitude, subjective norms, perceived behavioral control, and mass media reports. To identify new explanatory variables based on the TPB model, Brayley et al. (2015) combined the volunteer function inventory and the TPB model and determined, *via* multivariate regression analysis, that self-actualization predicted volunteer service behavior (Brayley et al., 2015). Manosuthi et al. (2020) combined the TPB model and norm activation model (NAM) to predict young volunteers' revisit. The results showed that the combination could increase the predictive ability to a certain extent. Before this, researchers not only applied the above two theories but also the theory of personal values is also added to explain how the behavioral intention of volunteer travelers is formed in the context of social activities. A longitudinal study on 230 volunteers in Italy used the TPB and role identity models to show that role identity fully mediates the relationship between behavioral intention, attitude, and subjective norms (Marta et al., 2014). Another study on the intention to participate in science volunteer services employed the expanded TPB model and showed that satisfaction is the only determining factor for sustained volunteering (Kao and Chien, 2019). Hence, TPB and its expanded model provide a suitable framework for understanding the intention to participate in health volunteer services. As it relates to the present study, when volunteers have positive attitudes toward floating population health volunteer services, they are more likely to demonstrate a willingness to participate. Similarly, when participation behavior matches individual and population norms, the individual is more likely to participate. Finally, when potential volunteers perceive that the services provided are easy, their willingness to participate will increase; otherwise, their willingness to participate will decrease. Therefore, the following hypotheses were proposed:

H1: Attitude positively affects willingness to participate in floating population health volunteer services.
H2: Subjective norm positively affects willingness to participate in floating population health volunteer services.
H3: Perceived behavioral control positively affects willingness to participate in floating population health volunteer services.

### Altruistic values

Altruism is an important component of the value system (Stern and Abel, 1999), and a person's behavior can be regarded as a function of altruistic values (Landon et al., 2018). The research shows that a person's empathy has a very strong and positive relationship with altruistic values, and individuals who mainly demonstrate altruistic behaviors often act in consideration of others for the promotion of harmonious social development. A study of 14 Australian non-profit organizations showed that values-congruency and altruistic motives best predict volunteer retention (Merrilees and Miller, 2020). Moreover, altruistic values are significant for the persistence of volunteering (Koo and Kim, 2012). In the health volunteer service domain, a measurement of the service motivation of 216 volunteers in an AIDS forum found that the relationship between motivation and altruism affects supportive behavior (Hu, 2020). In the travel volunteer industry, psychological resilience and personal norms are direct determinants of altruistic intentions (Han et al., 2020). Furthermore, the addition of a social justice function in the TPB model demonstrated the importance of intention to volunteer (Jiranek et al., 2013). Indeed, TPB affects prosocial behavior (i.e., organ donation, reducing environmental pollution, and consumption of organic foods), but altruism also appears to affect willingness to participate (Dennis and Buchholtz, 2009) through attitude, subjective norm, perceived behavioral control, and personality traits (Sanchez et al., 2018). Therefore, the following hypotheses were proposed:

H4: Altruistic values positively affect willingness to participate in floating population health volunteer services by volunteers.
H5: Altruistic values positively affect attitude in volunteers.
H6: Altruistic values positively affect subjective norms in volunteers.
H7: Altruistic values positively affect perceived behavioral control in volunteers.
H8: Altruistic values positively affect personality traits in volunteers.

### Personality traits

Personality traits, unique perceptual tendencies that support an individual's behavior (Zhou and Zhang, 2014), have also been used to predict intention to volunteer (Chen, 2017). Conscientiousness (a demonstration of ability) and agreeableness (a demonstration of cooperation) are two primary personality traits that are typically highlighted (McCrae, 1992; Paterson and Reniers, 2009; Omoto and Snyder, 2010; Claxton-Oldfield and Claxton-Oldfield, 2013). Carlo et al. (2005) examined five major personality traits to demonstrate the relationship between human personality traits and willingness to participate in volunteer services. Notably, results showed that as agreeableness decreased, the relationship between extraversion and motivation to volunteer increased (Carlo et al., 2005). A study on 261 counselors in a German volunteer service organization also showed that extraversion, neuroticism, and avoidance significantly affected beneficial behavior (Rek, 2016). In the same job, volunteers were happier and more extroverted than their paid counterparts (Elshaug and Metzer, 2001). Of course, in the hospice care industry, personality traits are more manifested as affinity and conscientiousness (Claxton-Oldfield and Claxton-Oldfield, 2013). Therefore, we proposed the following hypothesis:

H9: Personality traits positively affect willingness to participate in floating population health volunteer services in volunteers.

### External social incentives

The theory of the planned behavior model is not the only theoretical framework that examines the relationship between social behavior (i.e., volunteerism) and charitable behavior (Alias, 2015). While TPB includes both individual psychological factors and organizational stress factors, the effects of social factors on willingness to participate in health volunteer services must also be considered. Indeed, social factors may be critical to the motivation and ultimate mobilization of individuals to provide health services for the floating population. Social incentives, including material goods, honors, and career progression, have shown positive effects in other fields (i.e., voting turnout in Swedish voters; Funk, 2010). Similarly, in the volunteer service field, social incentives increase students' and community members' willingness to volunteer (Wang, 2003). Social incentives also directly affect the determination of values and emotional preferences of individuals toward volunteer services. Therefore, we proposed the following hypotheses:

H10: Social incentives positively affect willingness to participate in floating population health volunteer services.
H11: Social incentives positively affect attitude in volunteers.
H12: Social incentives positively affect subjective norms in volunteers.
H13: Social incentives positively affect perceived behavioral control in volunteers.

## Methods

### Data

Zhejiang Province is the second largest floating population in China and is also an economically developed province in the Yangtze River Delta. In this study, we identified Hangzhou, Ningbo, and Wenzhou in Zhejiang Province as representative cities with diverse immigrant populations, and the reason is that these three cities have a large internal floating population. Notably, Zhejiang Province has the second highest floating population in China and is an economically developed province in the Yangtze River Delta. The Ethics Committee of Wenzhou Medical University approved all data collection procedures. Before survey administration, a preliminary test was distributed (148/150 questionnaires were returned; recovery rate = 98%) to determine the consistency and reliability of the questionnaire (*via* Cronbach's alpha test and exploratory factor analysis). The revised questionnaire was distributed in June 2020 to individuals (aged 18–69 years; 1+ years of work experience) with a medical or healthcare industry background and proven experience in health volunteer services. To ensure that respondents had a medical background, the questionnaire was distributed only at community hospitals, medical universities, pharmaceutical companies, private clinics, and social organizations providing health services. In the sampling process, we took the annual floating population data of Zhejiang Province in 2017 as the sample frame. A stratified random population sampling method was used to randomly select three streets (towns) within the administrative area of each city, and 20–30 people with medical backgrounds were randomly selected from each street (town). Researchers trained investigators before the formal survey and provided on-site guidance and motivation when conducting the offline survey. Research investigators explained the aims, data collection methods, and survey instructions to participants before survey completion. The investigators also made clear that participation was anonymous and voluntary. Of the 780 questionnaires distributed, 770 were returned (recovery rate = 98.72%) and deemed valid.

### Measures

#### Willingness to participate

The willingness of participants with medical backgrounds to provide health services to the floating population was assessed *via* two questions: (1) “Are you willing to provide health volunteer services?” and (2) “Will you recommend volunteering in health services to others?” The 5-point Likert scale, which measures positive and negative responses to a specific statement, was used to measure each question (1 = totally disagree and 5 = totally agree).

#### Theory of planned behavior

The theory of planned behavior determined intention to volunteer *via* two different factors: attitude and subjective norm. In an effort to expand the applicability of the theory beyond individual control, perceived behavioral control was also added as a predictor to the TPB scale (Schifter, 1985; Ajzen, 2001). Hence, we studied whether attitude, subjective norm, and perceived behavioral control affected the willingness of an individual to participate in floating population health volunteer services. A 5-point positive scoring system was used (1 = totally disagree and 5 = totally agree). The attitude factors included four questions regarding the degree of awareness and degree of recognition and two variables. Subjective norm also included two variables (group pressure and self-restraint) and four questions. Perceived behavioral control included two variables (individual competency and convenience condition) and four questions.

#### Altruistic values

Questions from the Organizational Citizenship Behavior Checklist (Coyle-Shapiro, 2005) and the application of altruistic motivation measurements were also used to determine altruistic behavioral factors and decisions, respectively (Birch and Memery, 2018; Gim, 2019). Altruistic value measurements included cooperation tendency, self-improvement, and four questions. A 5-point positive scoring system was used (1 = totally disagree and 5 = totally agree), and the mean value was obtained. A higher score indicated greater altruistic motivation.

#### Personality traits

Emotions of normal people proposed the personality theory DISC in which human personality and behavior can be classified into four categories: dominance (D), influence (I), steadiness (S), and conscientiousness (C) (Marston, 2013). In the current study, each of these four indicators was used as a measurement variable in the development of four questions (Slowikowski, 2005). A 5-point positive scoring system was used (1 = totally disagree and 5 = totally agree) to analyze each of these variables.

#### Social incentives

According to Herzberg's dual-factor theory, hygiene factors are associated with materials and are mainly used to eliminate dissatisfaction. Although volunteer services are non-profit, the basic material needs of volunteers must be ensured. Satisfaction generated by motivational factors mainly includes extrinsic honors and social approval resulting from volunteer services (Herzberg, 2003). Based on this theory, three variables were analyzed (material incentive, spiritual incentive, and career progression) to create three measurement questions. A 5-point positive scoring system was used (1 = totally disagree and 5 = totally agree).

#### Control variables

In this study, sex, age, employment status, and education level were used as control variables.

#### Analysis strategy

In an effort to determine the roles of planned behavioral factors, altruistic values, personality traits, and social incentives on willingness to participate in health volunteer services, we employed structural equation modeling (SEM) to analyze the covariance matrix of these characteristic variables. SEM simultaneously processes multiple dependent variables, whereas the regression coefficient and path coefficients in conventional regression models are calculated independently for each dependent variable (effectively ignoring the effects of other variables). After obtaining model parameter estimations, SEM tested and evaluated the model. The primary objective was to test whether the proposed model was statistically significant as is or required adjustments. We used the AMOS 21.0 software to implement relevant fit test analyses and estimated relevant parameters *via* the maximum likelihood method. Sample size requirements of SEM dictate a sample ratio of between 1:5 and 1:10. In this study, 25 parameters were evaluated, so a sample size of at least 125–250 was required. From the validity rate of the returned questionnaires, the sample size satisfied SEM requirements. Bartlett's test shows that KMO <0.7, indicating that factor analysis can be carried out. Then, a total of seven common factors are proposed, and the cumulative sum of squares after rotation is >60%. After orthogonal rotation, the load of each item is >0.5. Therefore, it can be confirmed that the designed research scale has good construct validity.

## Results

The majority of participants were female (59%) and under the age of 30 (69%) (Table 1). As medical professionals, most participants reported completing several years of higher education (79.4%). The remaining 20.6% completed high school/technical secondary school, middle school, or primary school. The employment status of participants appeared to be relatively evenly distributed between students (34.7%) and medical company employees (35.5%); the remaining participants reported careers in hospital settings (14.2%), healthcare organizations (3.1%), or private clinics (8.8%). Table 2 shows the mean willingness to participate (mean = 2.68). Among planned behavioral variables, the attitude had the highest Likert score (mean = 3.71), followed by subjective norm (mean = 3.58), and perceived behavioral control (mean = 3.20), indicating that participants view the value of health volunteer services positively. Personality trait measures also received high scores (mean = 3.87), as did social incentive measures (mean = 3.72). Cronbach's alpha coefficient of each of the variables was >0.6, indicating good reliability between measures.

**Table 1:** Sample descriptive statistics.

**Marker**	**Type**	**Frequency**	**Percentage**
Sex	Male	316	41%
	Female	454	59%
Age	<18 years	56	7.3%
	18–30 years	474	61.6%
	30–40 years	167	21.7%
	40–60 years	69	9%
	>60 years	4	0.5%
Educational	Primary school and below	5	0.6%
level	Middle school	32	4.2%
	High school/technical secondary school	122	15.8%
	Junior college and above	611	79.4%
Employment	Student	267	34.7%
status	Medical company employee	273	35.5%
	Hospital employees	109	14.2%
	Healthcare organization staff	24	3.1%
	Private clinic	68	8.8%
	Others	29	3.8%

**Table 2:** Mean variable values and reliability analysis.

	**Mean**	**Standard**	**Cronbach's**
		**deviation**	**alpha**
Willingness to participate	2.68	1.08	0.907
Attitude	3.71	1.06	0.908
Subjective norm	3.58	0.86	0.907
Perceived behavioral control	3.20	1.03	0.910
Altruistic values	3.46	1.10	0.903
Social incentives	3.72	0.88	0.862
Personality traits	3.87	0.80	0.866

Prior to analyzing exploratory factors (Table 3), we used Bartlett's test of sphericity to determine that the overall correlation within our variables was significant (χ^2^ = 13,391.023, *p* = 0.00). The Kaiser-Meyer-Olkin measure of sampling adequacy was also high (KMO = 0.907), indicating that it was appropriate to proceed with factor analysis. We then utilized a principal component analysis to extract factors with eigenvalues >1 (Table 4). Seven common factors were identified, and the rotated cumulative sum of squares was 78.65% (Table 5). After orthogonal rotation, the 25 questions were classified between seven factors and the loading for each item was >0.5. Each observed variable was appropriately categorized according to the theoretical plan. Hence, the designed study scale had good structural validity.

**Table 3:** Exploratory factor analysis results.

**Marker**	**Component**						
	**1**	**2**	**3**	**4**	**5**	**6**	**7**
Willingness to participate (self-willingness)							0.827
Willingness to participate (recommendation of others)							0.839
Subjective norm (group pressure 1)		0.809					
Subjective norm (population pressure 2)		0.865					
Subjective norm (self-restraint 1)		0.793					
Subjective norm (self-restraint 2)		0.893					
Perceived behavioral control (individual capability 1)	0.870						
Perceived behavioral control (individual capability 2)	0.875						
Perceived behavioral control (convenience condition 1)	0.842						
Perceived behavioral control (convenience condition 2)	0.855						
Attitude (degree of awareness 1)				0.806			
Attitude (degree of awareness 2)				0.820			
Attitude (degree of recognition 1)				0.755			
Attitude (degree of recognition 2)				0.789			
Altruistic values (cooperation tendency 1)			0.826				
Altruistic values (cooperation tendency 2)			0.838				
Altruistic values (self-enhancement 1)			0.788				
Altruistic values (self-enhancement 2)			0.802				
Social incentives (allowance and subsidies)						0.850	
Social incentives (honors and awards)						0.828	
Social incentives (professional training)						0.803	
Personality trait (dominance)					0.788		
Personality trait (influencing)					0.758		
Personality trait (steadiness)					0.752		
Personality trait (conscientiousness)					0.843		
KMO	0.907
Bartlett's test of sphericity	13391.023 (*p =* 0.00)
Eigenvalue	9.141	2.504	2.414	2.02	1.32	1.248	1.015
Contribution to variance	12.87%	12.86%	12.78%	12.15%	11.99	9.471	6.535
Total contribution to variance	78.65%

**Table 4:** Confirmatory factor analysis results.

**Observed**		**Latent variable**	**Standardization**	**Residual error**	**T**	**P**	**CR**	**AVE**
**variable**			**factor loading**					
Q7	<–	Willingness to participate	0.902				0.906	0.829
Q6	<–	Willingness to participate	0.919	0.036	28.508	***		
Q11	<–	Subjective norm	0.930				0.909	0.715
Q10	<–	Subjective norm	0.756	0.029	27.134	***		
Q9	<–	Subjective norm	0.863	0.026	35.004	***		
Q8	<–	Subjective norm	0.822	0.028	31.732	***		
Q15	<–	Perceived behavioral control	0.873				0.911	0.719
Q14	<–	Perceived behavioral control	0.808	0.034	27.964	***		
Q13	<–	Perceived behavioral control	0.832	0.032	29.362	***		
Q12	<–	Perceived behavioral control	0.877	0.033	31.973	***		
Q19	<–	Attitude	0.888				0.908	0.712
Q18	<–	Attitude	0.811	0.029	28.944	***		
Q17	<–	Attitude	0.805	0.030	28.571	***		
Q16	<–	Attitude	0.867	0.030	32.568	***		
Q23	<–	Altruistic values	0.845				0.903	0.699
Q22	<–	Altruistic values	0.822	0.035	27.205	***		
Q21	<–	Altruistic values	0.827	0.036	27.466	***		
Q20	<–	Altruistic values	0.849	0.036	28.531	***		
Q27	<–	Social incentives	0.766				0.864	0.679
Q25	<–	Social incentives	0.871	0.048	23.648	***		
Q24	<–	Social incentives	0.832	0.047	22.993	***		
Q31	<–	Personality traits	0.860				0.869	0.625
Q30	<–	Personality traits	0.722	0.039	22.182	***		
Q29	<–	Personality traits	0.767	0.036	24.075	***		
Q28	<–	Personality traits	0.806	0.037	25.713	***		

**Table 5:** Discriminant validity.

	**Willingness to**	**Subjective**	**Perceived behavioral**	**Attitude**	**Altruistic**	**Social**	**Personality**
	**participate**	**norm**	**control**		**values**	**incentives**	**traits**
Willingness to participate	0.910						
Subjective norm	0.396**	0.845					
Perceived behavioral control	0.283**	0.272**	0.847				
Attitude	0.491**	0.431**	0.346**	0.843			
Altruistic values	0.472**	0.396**	0.270**	0.573**	0.836		
Social incentives	0.409**	0.283**	0.251**	0.336**	0.274**	0.824	
Personality traits	0.466**	0.308**	0.292**	0.421**	0.373**	0.511**	0.790

**Figure 1:**
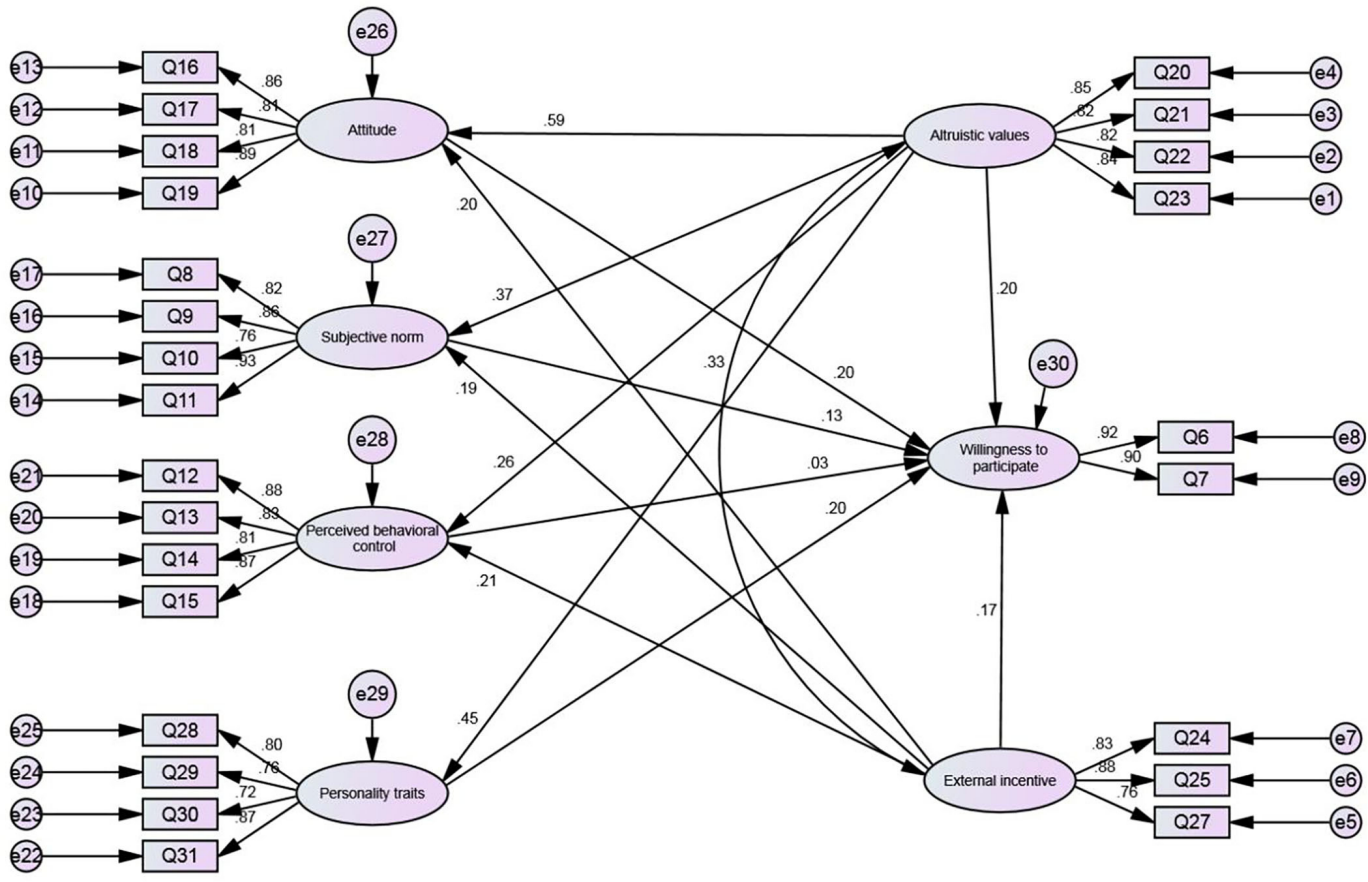
Path diagram of willingness to participate in floating population health volunteer services.

Figure 1 and Table 6 show the equation analysis results of the model. We constructed the corresponding structural equation according to the aforementioned theoretical model by using AMOS21.0 software to implement relevant fit test analyses. We used the maximum likelihood method to estimate related parameters and found that the model is a good fit (= 3.237).

**Table 6:** Hypothesis testing of Willingness to participate in floating population health volunteer services.

**Study hypothesis**			**Standardize**	**Non-standardized**	**Residual**	***T***	***P***	**Assumption**
			**coefficient**	**coefficient**	**error**			
H1: Attitude	→	Willingness to participate	0.204	0.190	0.043	4.414	***	Not rejected
H2: Subjective norm	→	Willingness to participate	0.128	0.143	0.041	3.493	***	Not rejected
H3: Perceived behavioral control	→	Willingness to participate	0.030	0.031	0.036	0.866	0.386	Rejected
H4: Altruistic values	→	Willingness to participate	0.204	0.198	0.051	3.871	***	Not rejected
H5: Altruistic values	→	Attitude	0.587	0.611	0.039	15.761	***	Not rejected
H6: Altruistic values	→	Subjective norm	0.374	0.325	0.034	9.551	***	Not rejected
H7: Altruistic values	→	Perceived behavioral control	0.261	0.245	0.038	6.421	***	Not rejected
H8: Altruistic values	→	Personality traits	0.448	0.353	0.031	11.438	***	Not rejected
H9: Personality traits	→	Willingness to participate	0.203	0.250	0.046	5.430	***	Not rejected
H10: Social incentive	→	Willingness to participate	0.172	0.231	0.051	4.493	***	Not rejected
H11: Social incentive	→	Attitude	0.197	0.283	0.050	5.640	***	Not rejected
H12: Social incentive	→	Subjective norm	0.195	0.235	0.047	4.962	***	Not rejected
H13: Social incentive	→	Perceived behavioral control	0.208	0.271	0.054	5.034	***	Not rejected

Attitude [β(*t* = 4.414, *p* < 0.001) = 0.204] and subjective norm [β(*t* = 3.493, *p* < 0.001) = 0.128] have significant positive effects on willingness to participate, whereas perceived behavioral control, on the other hand, does not have any effects on willingness to participate [β(*t* = 0.866, *p* = 0.386) = 0.030].

Altruistic values [β(*t* = 3.871, *p* = 0.00) = 0.204], attitude [β(*t* = 15.761, *p* = 0.00) = 0.587], subjective norm [β(*t* = 9.511, *p* = 0.00) = 0.374], perceived behavioral control [β(*t* = 6.421, *p* = 0.00) = 0.261], and personality traits [β(*t* = 15.761, *p* = 0.00) = 0.448] appear to have a significant positive effect on willingness to participate. Furthermore, personality traits have significant positive effects on willingness to participate [β(*t* = 5.430, *p* = 0.00) = 0.203].

Social incentives [β(*t* = 4.493, *p* = 0.00) = 0.172], attitude [β(*t* = 5.640, *p* = 0.00) = 0.197], subjective norm [β(*t* = 4.962, *p* = 0.00) = 0.195], and perceived behavioral control [β(*t* = 5.034, *p* = 0.00) = 0.208] also had significant positive effects on willingness to participate.

## Discussion and conclusion

Volunteer services provide peace of mind at challenging times (McGlinchey et al., 2022) and meet the typical needs of special populations that cannot be provided by traditional medical care services (Marcus, 2013). Meanwhile, volunteers have been proven to make great contributions to the health and well-being of the population. Although international research on volunteer services is extensive, research on health volunteer services in China is relatively underdeveloped. Studies on willingness to participate in floating population health volunteer services by medical professionals are particularly lacking. However, academic attention should be paid to health volunteer services as an important supplementation method for providing health services to the floating population. Hence, the current study referenced and expanded upon the TPB decision model to study the willingness to participate in floating population health volunteer services. Furthermore, while results from international studies provide a reference for volunteer service participation, China's social development, economic level, and cultural background differ from other countries and naturally cause differences in the factors affecting willingness to participate in health volunteer services.

The significant effect of attitude on willingness to participate in health volunteer services focused on two variables: degree of awareness and degree of recognition. Notably, when an individual demonstrates clear awareness of health volunteer services and understands the value of these services to the floating population (and society as a whole), they seem more willing to sacrifice time and effort for the benefit of others (Hayashi, 1984). When they realize that it is a responsibility, they are more willing to devote themselves to volunteering (Shi et al., 2018). To capitalize on this finding, the media could attract the attention of medical health professionals through pointed publicity in an effort to guide public opinion and increase awareness of the benefits of helping the floating population through health volunteer services. Let the professional population be more willing to participate in health volunteer services. Subjective norms, including group pressure and self-restraint, also affect participation in health volunteer services. Although volunteer services are voluntary behavior, China has a typical collectivist culture, family support, influence from friends, and recognition by the floating population *via* group pressure could significantly affect willingness to participate in health volunteer services (Wei and Wang, 2016). At the same time, there are no special rules for volunteer services and individual intention has a dominant position. If an individual has high self-actualization and a concept of repaying society, he/she demonstrates a greater willingness to participate in health volunteer services (Brayley et al., 2015). This is consistent with research results of Cho that when students have high self-development intentions, they will increase their willingness to volunteer (Cho and Jinkyu, 2020). Indeed, group motivation and self-improvement must be emphasized to increase the willingness of individuals to participate in health volunteer services. Interestingly, perceived behavioral control, including individual capabilities and external convenience, did not have significant predictive effects on health volunteer services, indicating that an individual's knowledge, experience, and other self-efficacies do not affect willingness to volunteer. This is contrary to the conclusion of Al Gharash and Hassan's previous research: knowledge and skills learned will increase the willingness and confidence of nursing students to provide volunteer services during the pandemic (Al Gharash et al., 2021). The main reason for this difference may be that China's current volunteer service organization for the floating population is still at the health education level and does not require high professional skills (Liu et al., 2019). Hence, professionals with medical backgrounds may feel as though nonprofessionals can carry out appropriate services. Therefore, individual capabilities would not have a significant value in health volunteer services, and they will naturally not care about whether the conditions for providing services are convenient. The effects of perceived behavioral control on willingness to participate must be reevaluated upon any transformation of the health volunteer services mission.

Altruistic values (including cooperation and self-enhancement) have significant predictive effects on willingness to participate in health volunteer services and indirectly affect willingness through attitude and subjective norms. In research, altruism value is regarded as a psychological reaction of volunteer tourists. Notably, individuals seem willing to provide service to others without the promise of a return as a manifestation of citizenship behavior. Reciprocal altruism may also play a role, where an individual's volunteer service is a manifestation of mutual assistance, and present efforts are exchanged for future benefits. Finally, reputation-oriented altruism, the “nice person” in traditional Chinese values, enhances the volunteer's reputation and individuals may be willing to participate in health volunteer services to maintain their own reputation (Deng et al., 2015). Similarly, altruistic values may cause individuals to develop positive attitudes toward health volunteer services and actively comply with the rules of the volunteer group. Therefore, the government and third-party organizations should actively cultivate altruistic values in people and include altruistic value education in national education systems. In particular, social responsibility courses should be included in medical education systems in hospitals and universities.

Personality traits also have significant predictive effects on willingness to participate in health volunteer services. People who are good at building good relationships with others and have good social skills may be more willing to participate in volunteering (Koo, 2019). Because of the voluntary nature of health volunteer services, individuals must decide for themselves whether to participate or not. Therefore, personality traits and associated psychological conflict aid in the decision-making process. To some extent, personality traits determine an individual's work performance (Il, 2019), and health volunteer services can be assigned based on personality traits. For example, individuals who display influence and dominance types of personalities are relatively open and may be more receptive to socializing or challenging work. Indeed, the future development of new floating population health services projects should be assigned to individuals with these personality traits. Individuals who demonstrate steadiness, on the other hand, have pleasant personalities and may be better suited for healthcare consultation services. Finally, individuals with conscientious personalities are often responsible people who are best suited for service supervision work. Overall, the management of volunteers should vary from person to person and attention should be paid to the personality characteristics of individuals so that assignment can be made for volunteers that best suits their personality (Bao et al., 2012).

Social incentives not only directly affect willingness to participate in health volunteer services but also indirectly affect willingness through attitude and subjective norms. If personal interests and community leaders are the initial reasons for volunteers to start volunteering, then additional work allowances and non-material rewards, such as community recognition and social prestige, are the main reasons for volunteers to stay (Afari-Asiedu et al., 2018). Participating in volunteer services requires external support. Hence, material incentives, spiritual incentives, and career development incentives may have positive effects on willingness to participate (Finkelstein, 2008). Although volunteer service is a form of dedicated behavior without the expectation in return, participants, nonetheless, sacrifice monetary resources and time. Therefore, necessary material incentives may ensure the continuous supply of healthy volunteers. The government is currently the main source of funding for health volunteer services, although volunteer service organizations also design outstanding health volunteer services to attract funding support from society. In addition, participants often hope to obtain recognition from others for their volunteer services and dedication (Wang et al., 2011). Therefore, providing some material support will increase volunteers' work productiveness and prevent volunteers from withdrawing due to financial limitations. Spiritual incentives, such as commendations and honorary titles, can also increase enthusiasm for participating in health services (Astray, 2008). Therefore, methods for linking volunteer services and personal development (i.e., converting service duration to points) should be explored. But, it is important to note that incentives are not mandatory for volunteers to feel that they participate in leisure activities and that they have a choice to accept or reject on their own terms (Stebbins, 2009).

In this study, we constructed a theoretical model on willingness to participate in floating population health volunteer services and validated this model. However, there are some limitations to this study. First, although the questionnaire used was a self-designed questionnaire based on mature scales with significant validity and reliability tests, there is no literature to date on the suitability of professional scales. The variables in this study were also based on variables extracted from the psychological decision model. There is a noticeable lack of studies on individual resource variables and environmental variables (i.e., financial status, social trust, media publicity, and governmental support) on willingness to participate in health volunteer services (Wu et al., 2018). China depends on governmental support as a prerequisite for the continuation of volunteer services. Therefore, the relationship between these factors and willingness to participate in volunteer services deserves further examination. Second, there were only two questions in the questionnaire that measure willingness to participate: (1) whether the individual is willing to participate and (2) whether the individual is willing to encourage others to participate. Hence, questions are lacking that analyze willingness to participate based on the type of volunteer service. Additionally, while there were many causative factors in this study model, we focused on the effects of these factors and did not specifically analyze each. Therefore, specific variables, such as how personality traits or altruistic values affect willingness to participate in volunteer services, should be explored in the future. Finally, in addition to the above approaches for expanding research on the research methods and variables, future works can also study the expansion of the scope and field, for example, should focus on group differences between participants (i.e., differences in willingness to participate between professional medical social workers, physicians, and medical university students), or other parts of China or the Chinese and foreign comparison, or study tourism volunteer service, cultural volunteer service, and other fields.

## Data availability statement

The raw data supporting the conclusions of this article will be made available by the authors, without undue reservation.

## Ethics statement

The studies involving human participants were reviewed and approved by the Ethics Committee of Wenzhou Medical University. Written informed consent from the patients/participants or patients/participants legal guardian/next of kin was not required to participate in this study in accordance with the national legislation and the institutional requirements.

## Author contributions

W-LW was conceived and designed the study and wrote the first draft of the manuscript, while HY-Y participated in the writing and critical improvement of the manuscript. Both of them were involved in measurement and data analysis. The article was rigorously revised, read and approved by all authors in the final manuscript.

## Funding

This work was supported by the National Social Science Fund of China (No. 17BZZ054).

## Conflict of interest

The authors declare that the research was conducted in the absence of any commercial or financial relationships that could be construed as a potential conflict of interest.

## Publisher's note

All claims expressed in this article are solely those of the authors and do not necessarily represent those of their affiliated organizations, or those of the publisher, the editors and the reviewers. Any product that may be evaluated in this article, or claim that may be made by its manufacturer, is not guaranteed or endorsed by the publisher.

## Abbreviations

TPB: Theory of Planned Behavior.
